# Pandemic preparedness: The case for sentinel surveillance of doctors’ burnout

**DOI:** 10.4102/jcmsa.v4i1.297

**Published:** 2026-01-28

**Authors:** Nondumiso Makhunga-Stevenson

**Affiliations:** 1 Ubuntu Doctor Coaching LLC, Elizabeth City, United States

**Keywords:** physician burnout, sentinel surveillance, pandemic preparedness, workforce resilience, South Africa, health systems, occupational health

## Abstract

Doctors’ wellbeing is an essential, yet often overlooked component of resilient health systems. Burnout, often described as the ‘canary in the coalmine’, offers a measurable, validated indicator of workforce strain that has direct implications for patient safety, quality of care and crisis response. This article argues for the integration of doctors’ burnout into sentinel surveillance frameworks as part of South Africa’s pandemic preparedness strategy. By embedding burnout monitoring within existing occupational health and surveillance systems, policymakers can generate actionable data, strengthen workforce resilience, and safeguard system performance during future epidemics.

## Introduction

Doctors’ wellbeing is a critical determinant of health system resilience. There is evidence linking the impact of doctors’ wellbeing to patient safety, quality of care, workforce stability, financial resilience of healthcare organisations^1^ and effectiveness of pandemic and crisis responses.^2^ The wellbeing of doctors and other healthcare workers is often poorly measured, representing a missed opportunity to strengthen quality, and establishing a vital metric for health system performance and resilience.^3^ Without urgent action to routinely measure doctors’ burnout, South Africa risks entering the next pandemic with exhausted doctors, high attrition and systemic collapse.

While a comprehensive framework for doctors’ wellbeing should include both negative indicators (such as burnout and moral injury) and positive indicators (such as meaning, engagement and thriving), burnout offers a practical and validated ‘canary in the coalmine’.^3,4^ Wellbeing is typically framed as an individual responsibility of the doctor and reduced to personal wellness programmes. For resource-constrained countries such as South Africa, starting with burnout as a measurable entry point is both feasible and urgent and lays the groundwork for broader wellbeing metrics that capture the full spectrum of doctors’ resilience.

While doctor-specific wellness programmes show promise, they are often under-resourced, under-utilised, lack scientific rigour, and have not resulted in meaningful changes in clinical burnout.^5^ With piecemeal wellness programmes but no surveillance, burnout remains costly.

This article argues that sentinel surveillance of doctors’ burnout in South Africa is not only feasible within the existing occupational and public health infrastructure, but it is also necessary to elevate doctors’ wellbeing as a public health imperative to protect health workers and strengthen the health system’s resilience. Sentinel surveillance can shift the focus towards early detection of population-level trends, identify hotspots or rising burnout rates before they escalate, and enable timely, data-driven and proportionate responses. Data collected from such surveillance could guide strategies that create conducive work environments, for example, addressing staff shortages, workload management and change in the institutional culture.

Burnout lends itself well to surveillance as it meets established criteria; clear case definitions, direct measurement tools (e.g. Maslach Burnout Inventory),^6^ and proxy measures (e.g. absenteeism and turnover rates).^7^ These features make burnout both measurable and actionable, positioning it as a legitimate target for routine occupational health surveillance.

South Africa’s context makes the case for burnout surveillance especially compelling. Like many sub-Saharan countries, its health system operates under budget constraints, which limit investments in staffing, infrastructure, and wellness programmes, underscoring the need for structured monitoring and early response.

## Burnout as an occupational health phenomenon

The World Health Organization (WHO) defines burnout^8^ as ‘a syndrome resulting from chronic, unmanageable workplace stress that has not been successfully managed’ emphasising its link to the workplace. Burnout is characterised by three components: emotional exhaustion, depersonalisation or cynicism, and decreased sense of accomplishment and professional efficacy.

As an occupational health phenomenon, the legal, professional and ethical responsibility to safeguard the mental health and wellbeing of healthcare workers lies primarily with policymakers and employers. The coronavirus disease 2019 (COVID-19) pandemic unmasked the systemic failure to protect the individual healthcare workers from trauma, excessive workload and challenging work conditions, while simultaneously exposing the need for alignment of evidence, policy and practice in tackling burnout. This is evidenced by extensive research documenting high levels of burnout among South African doctors and medical students.^9,10,11^ Research efforts on the impact of burnout remain episodic and lack a central coordinating mechanism to translate findings into actionable outcomes. This fragmentation underscores the necessity of reframing burnout not merely as an episodic phenomenon but as an upstream proxy and early warning signal for potential crises including mental health risks such as depression and suicide.

## Burnout as an upstream proxy

While burnout and depression are distinct conditions, the relationship between burnout, depression and suicide is complex. Studies in both high-income and low-and-middle-income countries provide evidence that burnout is associated with depression and increased suicidal ideation among doctors and medical students.^12^

Limitations in occupation specific cause-of-death data likely result in under-reporting of suicide among doctors, yet reported data signal significant levels of suicide, with female doctor suicide deaths 250% – 400% higher than the general female population in some studies.^13^ In this context, burnout offers potential for a pragmatic proxy for early detection and to prevent downstream sequelae such as major depressive disorder, generalised anxiety disorder, suicidal ideation and to prevent mortality.^14^

## The surveillance gap in South African policy

A cursory review of key national strategic documents such as the National Department of Health Strategic Plan 2025–2030, the Second Presidential Health Compact (2024–2029), and the National Department of Health Annual Performance Plan 2025–2026, which all prioritise human resources for health, reveals no explicit provision for distress indicators or for systematic measurement of healthcare workers’ wellbeing. Across these documents, healthcare workers are consistently framed as system inputs to achieve service coverage, and not as professionals who require protection from a system that contributes to burnout. While the COVID-19 pandemic has created a level of institutional awareness, the failure to capitalise on the momentum with concrete systemic solutions risks normalising chronic burnout, moral injury and making healthcare worker wellbeing invisible. This constitutes a major gap, given known links between workforce wellbeing, retention, patient safety and system resilience. Proactively incorporating burnout surveillance indicators into the foundational architecture of these strategic documents would not only protect health workers but also strengthen patient safety, accelerate progress towards universal health coverage, improve accountability and facilitate data-driven decision-making.

## International trends in burnout surveillance

By contrast, high-income countries are moving towards adopting system-level approaches. Their models suggest advances in testing how doctors’ burnout surveillance can be incorporated into national health monitoring systems. In the United States, the National Plan for Health Workforce Well-Being^15^ provides for a systems-based, multilevel approach with the frontline as the entry point, which not only aims to systematically assess and monitor health worker burnout and wellbeing but also provides data on disparities, guides resource allocation and evaluates the effectiveness of interventions across diverse clinical and public health settings.

Other initiatives, such as Network on the Coordination and Harmonisation of European Occupational Cohorts (OMEGA-NET),^16^ have sought to harmonise and consolidate evidence for the joint development of a funded research agenda to advance preventive occupational health policies.

While some are nascent, these international models demonstrate that doctor-specific monitoring can generate data that can potentially be triangulated with service-use records, mortality registries, and periodic burnout surveys. South Africa risks falling behind global trends in health workforce wellbeing surveillance if it does not act, but has a strategic opportunity to lead the Global South by piloting scalable models of burnout surveillance, thereby contributing to international benchmarks.

Emerging technological applications and advances in artificial intelligence (AI)-enabled analytics hold the potential to complement existing surveillance tools for burnout surveillance and could evolve into a real-time ‘early warning system’ for health system resilience.

## A framework for action

While international models are instructive, a South African framework must be tailored to its unique health system architecture and constraints. A consultative process involving doctors, professional societies, training institutions, and policymakers would be a significant step towards building broad ownership and consensus on sustainable surveillance. Notwithstanding the aforesaid, this article proposes three ‘quick-win’ actions as proof of concept to provide a pathway from principle to practice and to signal urgency and commitment.

### Recommendations

- **Embed burnout and other wellbeing metrics into accreditation standards for healthcare facilities and training institutions:** Institutions mandated to protect doctors’ health and safety, for example, hospitals and academic institutions could integrate these metrics into quality frameworks, and postgraduate training evaluations to ensure accountability and wellbeing becomes a non-negotiable metric of institutional performance.
- **Establish a network for a national research agenda on burnout and healthcare worker wellbeing:** Academic institutions and professional societies collaborate to establish a central platform to harmonise data, support evidence-based policies, maximise the impact of research and mobilise resources in a coordinated manner. This will ensure that the conversation is driven by robust, South African-specific data.
- **Develop pilot sites at facility level for burnout sentinel surveillance in diverse context:** This provides the proof of concept needed to build political and financial buy-in. Selected facilities should represent a diversity of settings, for example, urban, rural, academic, private and public health facilities.

Together, these initiatives would create a multifaceted system of accountability, data generation and practical learning, moving beyond isolated studies towards integrated, system-level monitoring.

## Conclusion

### A call to action

Sentinel surveillance of doctors’ burnout represents a paradigm shift. It holds promise for implementation of timely interventions that protect both doctors and patients, facilitation of structural evidence-based reforms in the workplace and building of a system resilient enough for current demands and future epidemic preparedness. Institutionalising burnout surveillance is therefore essential to pandemic preparedness and the protection of South Africa’s doctors. Without urgent action, South Africa risks entering the next pandemic with vulnerabilities that jeopardise both health workers and the public they serve.
